# Exerkines and Sarcopenia: Unveiling the Mechanism Behind Exercise-Induced Mitochondrial Homeostasis

**DOI:** 10.3390/metabo15010059

**Published:** 2025-01-16

**Authors:** Jiayin Wang, Dandan Jia, Zhiwang Zhang, Dan Wang

**Affiliations:** 1 School of Exercise and Health, Shanghai University of Sport, Shanghai 200438, China; wjy@sus.edu.cn (J.W.); jiadandan@sus.edu.cn (D.J.); 2 School of Athletic Performance, Shanghai University of Sport, Shanghai 200438, China

**Keywords:** exercise, exerkines, sarcopenia, mitochondrial homeostasis

## Abstract

**Background/Objectives**: Sarcopenia, characterized by the progressive loss of muscle mass and strength, is linked to physical disability, metabolic dysfunction, and an increased risk of mortality. Exercise therapy is currently acknowledged as a viable approach for addressing sarcopenia. Nevertheless, the molecular mechanisms behind exercise training or physical activity remain poorly understood. The disruption of mitochondrial homeostasis is implicated in the pathogenesis of sarcopenia. Exercise training effectively delays the onset of sarcopenia by significantly maintaining mitochondrial homeostasis, including promoting mitophagy, improving mitochondrial biogenesis, balancing mitochondrial dynamics, and maintaining mitochondrial redox. Exerkines (e.g., adipokines, myokines, hepatokines, and osteokines), signaling molecules released in response to exercise training, may potentially contribute to skeletal muscle metabolism through ameliorating mitochondrial homeostasis, reducing inflammation, and regulating protein synthesis as a defense against sarcopenia. **Methods**: In this review, we provide a detailed summary of exercise-induced exerkines and confer their benefit, with particular focus on their impact on mitochondrial homeostasis in the context of sarcopenia. **Results**: Exercise induces substantial adaptations in skeletal muscle, including increased muscle mass, improved muscle regeneration and hypertrophy, elevated hormone release, and enhanced mitochondrial function. An expanding body of research highlights that exerkines have the potential to regulate processes such as mitophagy, mitochondrial biogenesis, dynamics, autophagy, and redox balance. These mechanisms contribute to the maintenance of mitochondrial homeostasis, thereby supporting skeletal muscle metabolism and mitochondrial health. **Conclusions**: Through a comprehensive investigation of the molecular mechanisms within mitochondria, the context reveals new insights into the potential of exerkines as key exercise-protective sensors for combating sarcopenia.

## 1. Introduction

Sarcopenia, a geriatric syndrome marked by diminishing muscle mass and strength, has become increasingly prevalent in recent years [1]. It is frequently associated with negative physical and metabolic changes, leading to a decreased quality of life, increased mortality rates, and higher healthcare costs [2], all further exacerbated by the COVID-19 pandemic [3]. The prevalence of sarcopenia among the global elderly population varies between 10% and 16%, depending on the classification criteria and cut-off points used [4]. Developing effective sarcopenia interventions is essential to extend life span [5]. Despite the lack of specific medications approved for sarcopenia treatment, there is a consensus that physical activity is the only validated intervention [1,6,7]. Evidence-based clinical guidelines strongly advocate for regular exercise or physical activity, or exercise training as the main approach to managing sarcopenia [8].

Engaging in regular physical activity or exercise training, which proves beneficial for enhancing mitochondrial homeostasis in individuals with sarcopenia, significantly regulates mitophagy, mitochondrial biogenesis, mitochondrial dynamics, and mitochondrial redox biology [9,10,11]. However, few studies have explored the molecular mechanisms underlying exercise-induced mitochondrial homeostasis in sarcopenia. A crucial aspect involves exercise-inducible exerkines, comprising myokines, hepatokines, osteokines, and adipokines [12,13]. In this review, we will provide a detailed examination of the signaling pathways in different types of exercise intervention and mitochondrial homeostasis for preventing sarcopenia. Following this, we will focus on addressing exerkine-mediated mitochondrial homeostasis in combating sarcopenia. The understanding of these molecular mechanisms underlying exerkines’ benefits for skeletal muscle health will help promote exercise training in both healthy individuals and those with sarcopenia, as well as advance the development of the key protective sensors of exercise as novel therapeutic targets for managing this condition.

## 2. Management and Therapeutic Approaches for Sarcopenia

### 2.1. The Beneficial Impact of Exercise on Sarcopenia

Physical inactivity can lead to a decrease in skeletal muscle protein synthesis, lean tissue mass, and extremity strength, contributing to the increased prevalence of sarcopenia [14]. In contrast, regular training and daily activity are effective countermeasures to promote hormonal balance, reduce oxidative stress, improve mitochondrial dysfunction, and influence immune function [15]. Furthermore, numerous studies have shown that the prevention and management of sarcopenia by exercise training are related to the type and intensity of exercise [16]. Considerable focus has been directed towards exploring the effects of exercise intervention on skeletal muscle, targeting resistance (strength and power) [17], aerobic [18], and high-intensity interval work [19]. Different types of exercise enhance various aspects of physical function and can be combined as needed, allowing for a customized, multicomponent intervention.

**Figure 1:**
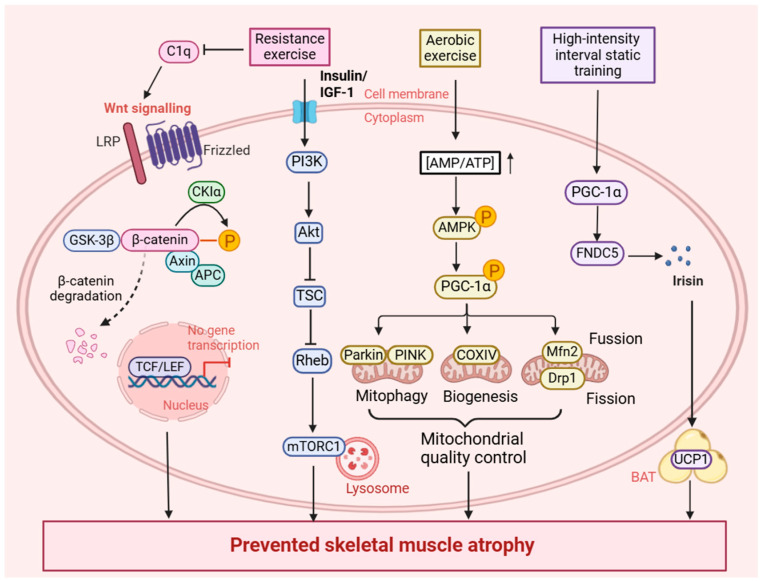
Exercise alleviates sarcopenia by regulating multiple signaling pathways. Resistance training prevents muscle fibrosis and atrophy via down-regulation of C1q-induced Wnt signaling or the PI3K-Akt-TSC signaling cascade to regulate mTORC1 signaling through Rheb, which subsequently translocates to lysosomes. Aerobic exercise promotes an increase in the intracellular AMP/ATP ratio, subsequently activating the AMPK/PGC-1α signaling pathway to promote mitochondrial quality control. PGC-1α may activate other transcriptional partners including NRF-1/2 and ERR, which subsequently drive the downstream transcription of genes involved in mitochondrial biogenesis. PGC-1α could enhance the Beclin1 and LC3-II/LC3-I ratio and decreases p62 to significantly increase mitophagy flux. The overexpression of PGC-1α also helps counteract age-related decline in mitochondrial fusion and fission biomarkers such as Mfn2 and Drp1. High-intensity interval static strength training upregulated PGC-1α expression in skeletal muscle, which facilitated the restoration of mitochondrial function. PGC-1α promotes the expression of FNDC5, leading to elevated circulating levels of irisin. Additionally, the increased irisin levels subsequently enhanced the expression of UCP1, thereby stimulating the conversion of white fat into brown fat. PI3K, phosphoinositide 3-kinase; Akt, protein kinase B; TSC, tuberous sclerosis complex; Rheb, Ras homolog enriched in brain; mTORC1, mechanistic target of rapamycin complex 1; C1q, complement component 1q; CKIα, casein kinase Iα; GSK3β, glycogen synthase kinase -3β; TCF, T-cell factor; LEF, lymphoid enhancer-binding factor; PGC-1α, peroxisome proliferator-activated receptor-γ coactivator-1α; FNDC5, fibronectin type III domain containing 5; UCP1, uncoupling protein 1; AMPK, adenosine monophosphate-activated protein kinase; COXIV, cytochrome oxidase subunit IV; Drp1, dynamin-related protein 1; Mfn2, mitofusin 2; P, phosphorylation; BAT, brown adipose tissue.

An experiment utilized three-month-old mice as models for sarcopenia, which were subjected to lifelong moderate-intensity treadmill running at 12 m/min for 55 weeks [18]. The results demonstrate that lifelong aerobic exercise activates the AMPK/PGC-1α signaling pathways, which helps counteract the age-related decline in mitochondrial fusion and fission biomarkers, enhances the Beclin1 and LC3-II/LC3-I ratio and decreases p62, and effectively promotes mitochondrial biogenesis, thereby improving mitochondrial quality control and delaying the onset of sarcopenia [18]. According to a previous study, resistance training in mice (three times a week for 12 weeks, climbing ladders) prevents muscle fibrosis and atrophy by down-regulating complement component 1q (C1q)-induced Wnt signaling (glycogen synthase kinase-3β/β-catenin), including β-catenin in satellite (Pax7/DAPI) and fibroblast (vimentin/DAPI) cells [17]. And resistance training may activate the PI3K-Akt-TSC signaling cascade to regulate mTORC1 signaling through Ras homolog enriched in brain (Rheb), which subsequently translocates to lysosomes, thereby playing a role in promoting protein synthesis by activating several translational and ribosomal components in skeletal muscle hypertrophy [20] (Figure 1).

A study demonstrated that a regimen consisting (totally 30 weeks) of 2 d/week high-intensity resistance training (three sets × 8–12 repetitions to failure plus three sets of the same abdominal flexion exercise), inserting an additional weekly session of low-load, explosive resistance training (resistance loads were only two-thirds of the loads prescribed on high-intensity resistance training), significantly decreased the expression of pro-inflammatory cytokine receptors and maximized thigh muscle mass and overall lean body mass in older adults [21]. Moreover, compared with sedentary mice, those undergoing 10-min uphill treadmill, high-intensity interval training (HIIT) sessions over 16 weeks showed significant improvements in grip strength, treadmill endurance, and gait speed, and exhibited increased muscle mass, larger muscle fiber size, and greater mitochondrial biomass [19]. Additionally, an experiment showed that a high-intensity interval static strength training (eight weeks) program in aged rats increased the muscle fiber volume, prevented atrophy, and improved motor function via the PGC-1α/FNDC5/UCP1 signaling pathway, leading to elevated circulating levels of irisin and subsequently stimulating the conversion of white fat into brown fat (Figure 1) [22]. A review of the extant literature indicates that all three types of exercise are effective in managing sarcopenia. However, a definitive study is required to determine which one is superior. This is a promising avenue for future research. Specificity is a fundamental principle in exercise training. Hence, the exercise dosage should be adjusted in clinical practice based on the individual progress of patients with sarcopenia. Furthermore, the key training variables (exercise type, intensity, duration, and frequency) should be combined and tailored until the optimal dosage for each individual is identified. And the correlation of the main variables with the reduction in sarcopenia events requires further investigation.

### 2.2. The New Potential Medical Treatments on Sarcopenia

Pharmacotherapy represents an effective approach to managing sarcopenia in the elderly, particularly when exercise interventions alone are insufficient. Older adults with conditions that impair energy supply or digestive function, such as cancer cachexia, often present with exercise intolerance and reduced exercise capacity, which restricts the potential efficacy of exercise therapy [23].

Recent studies have shown that mitochondria-derived peptides (MDPs) play a key role in regulating muscle homeostasis and preventing sarcopenia [24]. The MDPs that have been the focus of intensive research in recent years are primarily MOTS-c (mitochondrial open reading frame of the 12S ribosomal RNA type-c) and humanin. MOTS-c administration enhances muscle glucose uptake, boosts exercise endurance, and prevents muscle atrophy by directly binding and activating casein kinase 2 (CK2) [25]. Humanin administration promotes autophagy and reduces the accumulation of abnormal proteins by activating AMPK upstream of PI3K Class III complex formation, thereby enhancing skeletal muscle health and function [26]. Additionally, therapeutic strategies targeting pro-inflammatory cytokines, particularly TNF-α, are gaining recognition as potential approaches to mitigate sarcopenia. For instance, etanercept, a TNF-α inhibitor, has demonstrated efficacy in preventing muscle atrophy and extending lifespan in aged mice through mechanisms including the preservation of muscle fiber cross-sectional area and type II muscle fibers [27]. These findings have shown promising results in clinical trials, suggesting potential improvements in muscle mass and strength.

## 3. Exercise Mitigates Sarcopenia by Maintaining Mitochondrial Homeostasis

**Figure 2:**
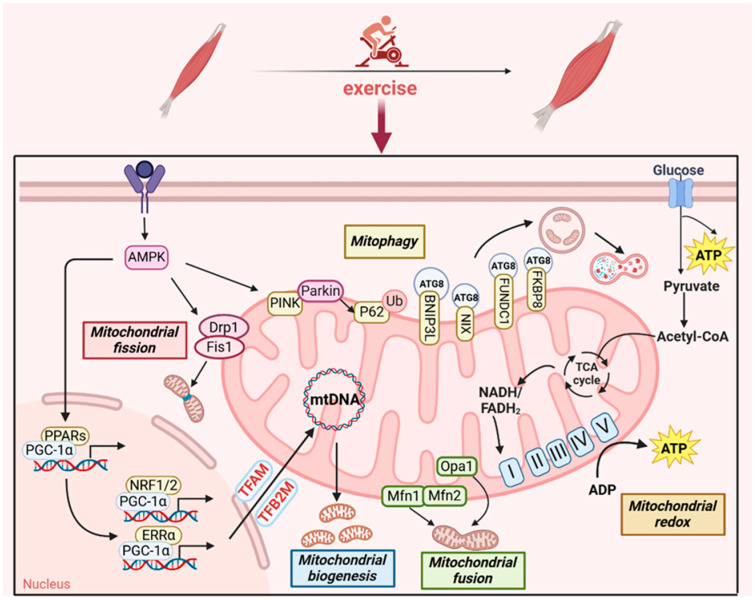
Exercise promotes mitochondrial homeostasis in sarcopenia. Regular exercise triggers AMPK signaling, leading to PGC-1α upregulation and activation. PGC1-α may form a transcriptional regulatory complex with PPARs to coactivate other transcriptional partners, such as NRF1/2 and ERRα, involved in mitochondrial biogenesis. Exercise-induced glucose catabolism is implicated in regulating mitochondrial redox to yield ATP. Exercise can also activate Parkin-dependent and ubiquitin-independent mitophagy to attenuate the impact of aging on muscle tissue. The fusion of the outer mitochondrial membrane is mediated by Mfn1/2, and Opa1 promotes the fusion of the inner mitochondrial membrane, together triggering mitochondrial fusion. Mitochondrial fission of the outer mitochondrial membrane is mediated by Drp1 and Fis1. PGC-1α, peroxisome proliferator-activated receptor-γ coactivator-1α; NRF1/2, nuclear respiratory factors 1 and 2; ERRα, estrogen-related receptor alpha; PPARs, peroxisome proliferator-activated receptors; TFAM, mitochondrial transcription factor A; TFB2M, mitochondrial transcription factor B2; mtDNA, mitochondrial DNA; AMPK, AMP-activated protein kinase; ATG8, autophagy-related protein 8; TCA, tricarboxylic acid cycle; Drp1, dynamin-related protein 1; Fis1, mitochondrial fission protein 1; Mfn1/2, mitofusin1/2; Opa1, optic atrophy 1; NADH, nicotinamide adenine dinucleotide; FADH, flavine adenine dinucleotide.

Mitochondria have not only been considered energy suppliers but have also been closely studied in sarcopenia, observed to play a role in cell signaling, Ca^2+^ homeostasis, and cell death [28]. However, mitochondria possess unique DNA (mtDNA), with replication rules differing from genomic DNA. The production of endogenous reactive oxygen species (ROS) by mitochondria has traditionally been accepted as an uncontrolled process that triggers oxidative stress, causing large numbers of mtDNA mutations and damage to mitochondrial membrane proteins [29]. Under pathological conditions, a decreased mitochondrial calcium uptake 1 (MICU1)/mitochondrial calcium uniporter (MCU) ratio results in a rapid influx of cytoplasmic Ca^2+^ into the mitochondria, leading to mitochondrial Ca^2+^ overload [30]. Elevated mitochondrial Ca^2+^ levels interact with phosphate compounds, resulting in damage to the electron transport chain and the subsequent generation of ROS [31]. Excessive mutations in ROS-producing mtDNA result in mitochondrial abnormalities, influencing aspects such as mitophagy, mitochondrial biogenesis, and dynamics, and inducing sarcopenia and alleviated physical activity or exercise training [32]. A recent study demonstrated that treatment with the mitochondrial ROS scavenger mito-TEMPO prevents unloading-induced mitochondrial dysfunction and helps preserve fatigue resistance in the soleus muscle in rats [33]. Hence, protecting both the quantity and quality of mitochondria is of vital importance for combating sarcopenia through maintaining mitochondrial homeostasis (Figure 2) [34].

### 3.1. Exercise Induces Mitophagy in Sarcopenia

Mitophagy is the process by which damaged mitochondria are selectively removed through autophagic degradation [35]. Loss of Parkin, a mitophagy protein, results in reduced muscle strength, impaired mitochondrial respiratory, and an increased sensitivity of skeletal muscle to mitochondrial permeability transition pore (mPTP) opening in mice [36]. Conversely, Parkin overexpression not only reduces aging-related increases in markers of oxidative stress, fibrosis, and apoptosis, but also enhances mitochondrial content and enzymatic activities, mitigating sarcopenia and improving skeletal muscle performance [37]. The PTEN-induced oxidation kinase protein 1 (PINK1)/Parkin pathway has been widely studied [38]. When mitochondrial membrane potential is compromised, the transport of PINK1 into the inner mitochondrial membrane (IMM) is inhibited, leading to its accumulation on the cytoplasmic surface of the outer mitochondrial membrane (OMM). This subsequently activates Parkin, causing a conformational change that transforms Parkin into activated E3 ubiquitin ligase, which will ubiquitinate mitochondrial proteins [39]. It should be noted that beyond ubiquitin-dependent mitophagy, other forms are also relevant, such as ubiquitin-independent mitophagy, which mainly depends on outer mitochondrial membrane-resident mitophagy receptors including BNIP3L/NIX, FUNDC1, and FKBP8 in mammals [40].

When damaged mitochondria are not eliminated, apoptotic signaling pathways are triggered to damage the nucleus, which can activate autophagy and ubiquitin ligases to mediate protein degradation. Resistance exercise (ladder-climbing training, 3 days/week for 9 weeks) has been shown to enhance autophagy and reduce apoptosis in the skeletal muscle of older rats by modulating insulin-like growth factor-1 (IGF-1) and its receptors, as well as the downregulation of Akt/mTOR and Akt/FOXO3a signaling pathways [41]. Previous studies have shown that acute treadmill running (intervals of 10 min at 13 m/min, 10 min at 16 m/min, 50 min at 19 m/min, and 20 min at 21 m/min) in mice causes mitochondrial oxidative stress within 3 to 12 h, and mitophagy at 6 h post-exercise via the Ampk-Ulk1 signaling pathway, providing clear evidence of exercise-induced mitophagy [42]. Likewise, acute exercise (90 min) has been observed to result in enhanced mitophagy and an enhanced targeting of mitochondrial degradation, increasing mitochondrial turnover [43]. This outcome is at least partially coordinated by PGC-1α, as the results were not replicated in PGC-1α knockout (KO) mice. Remarkably, a 12-week resistance exercise (ladder-climbing training, 3 days/week) program in rats with sarcopenia downregulated the expression of E3 ubiquitin ligases, including atrogin-1 and MuRF1, in skeletal muscle tissue through the AMPK/FoxO3 signaling pathway, promoting mitophagy and enhancing mitochondrial function, as well as suppressing skeletal muscle atrophy [44]. In addition, it has been reported that after 5 weeks of swimming training, mice exhibited increased levels of basal mitophagy in vivo, as indicated by a higher LC3-II/LC3-I ratio and elevated BNIP3 levels in the mitochondrial fraction, thereby contributing to skeletal muscle adaptation and enhanced muscle strength [45]. Mitophagy is increasingly recognized as a crucial mechanism in exercise-induced remodeling, but data from human studies on this topic remain limited. Further investigation is required to fully elucidate the mechanism of mitophagy in humans. The potential of mitophagy to alleviate sarcopenia represents a promising avenue for future research.

### 3.2. Exercise Induces Mitochondrial Biogenesis in Sarcopenia

Mitochondrial biogenesis is the process through which cells generate new, normally functioning mitochondria [46]. The process consists of multiple transcription factors that regulate the expression of nuclear genes responsible for encoding mitochondrial proteins, such as the nuclear respiratory factors 1 and 2 (NRF1/2), peroxisome proliferator-activated receptors (PPARs), and the estrogen-related receptor alpha (ERRα), which bind to promoters and activate gene transcription [47]. In addition, PGC-1α, regulated by exercise training, serves as a pivotal transcriptional regulator in mitochondrial biogenesis, impacting muscle morphology and physiological function [48,49]. Overexpression of PGC-1α increases mitochondrial protein content and antioxidant enzyme activity, in addition to modifying gene expression, thereby attenuating the impact of aging on muscle tissue [50,51].

Muscle exercise increases the production of ROS (e.g., hydrogen peroxide (H_2_O_2_)), leading to oxidative stress in many tissues, including skeletal muscle [52]. Researchers have found that after H_2_O_2_ treatment, skeletal muscle cells can lower ATP levels, activate AMPK, and increase PGC-1α mRNA, suggesting that H_2_O_2_ can promote PGC-1α expression by AMPK, finally accelerating mitochondrial biogenesis [53]. MicroRNAs (miRNAs) are a category of endogenous small RNAs governing gene expression at the post-transcriptional level. Recently, a study summarized and analyzed that just eight weeks of treadmill training in mice enhances the activity of peroxisome proliferator-activated receptor delta (PPARδ), which in turn activated the Lin28a/miRNA let-7b-5p pathway in the skeletal muscle, leading to changes in mitochondrial metabolism in a PGC-1α-dependent manner [54]. And 12-week aerobic exercise (treadmill running, 17.5 m/min for 45 min/day) could mitigate the aging-associated decline in PGC-1α and mitochondrial transcription factor A (TFAM) in aged rats, resulting in expression levels that are even higher than those observed in untrained young rats [55]. Hence, the elevation of PGC-1α and TFAM, and the subsequent augmentation of mitochondrial biogenesis, may be key mechanisms through which exercise contributes to the preservation of mitochondrial quality in skeletal muscle.

### 3.3. Exercise Induces Mitochondrial Dynamics in Sarcopenia

Mitochondria, as highly dynamic organelles, engage in a continuous process of fusion and fission to uphold their morphology and functionality, collectively known as mitochondrial dynamics. This process is essential for regulating muscle stem cell regeneration by regulating metabolism, protein balance, and mitophagy for regenerative therapies in sarcopenia [56]. It is crucial to maintain a balance between these two processes to ensure the efficient functioning of both mitochondria and skeletal muscle. Nevertheless, mitochondrial abnormalities are common features of sarcopenia with aging, including disruptions in the proteins responsible for mitochondrial fusion and fission, such as mitofusin1/2 (Mfn1/2) that mediates OMM fusion, optic atrophy 1 (Opa1) that mediates IMM fusion, dynamin-related protein 1 (Drp1), and mitochondrial fission protein 1 (Fis1) [57]. Several studies have demonstrated that aging leads to an increased occurrence of mitochondrial fission, which aligns with the fragmented mitochondrial structures commonly observed in the skeletal muscle of aged rodents [58]. Congruently, Opa1 levels decline with age in sedentary but not active adults, which is associated with muscle mass, inflammation, and metabolic homeostasis in muscle [59].

Mfn2 deficiency in aging muscle worsens age-related mitochondrial dysfunction, underlying the age-related alterations in metabolic homeostasis and sarcopenia [60]. Opa1 deficiency reduces muscle fiber size, causes mitochondrial dysfunction and mitochondrial DNA release, and enhances FGF21, characterized by NF-κB activation, which contributes to muscle loss and weakness [61]. The mitochondria-related proteins Drp1, Mfn2, PGC-1α, and COX significantly increased in mice through Sestrin2 in an AMP-activated protein kinase α2 (AMPKα2)-dependent manner following an 8-month treadmill training (60 min/day, 12 m/min) program, suggesting that aerobic exercise enhanced the mitochondrial fusion and fission in gastrocnemius with age-related sarcopenia [62]. It is well established that resistance exercise can increase muscle protein synthesis and muscle strength. In rat skeletal muscle, there was no change in mitochondrial protein levels involved in mitochondrial fission or fusion over the 24 h after acute resistance exercise, while four weeks of electrical stimulation-induced resistance training (every other day, 12 sessions) increased Mfn1, Mfn2, and Opa1 protein levels [63]. The variations in study results could be attributed to differences in training protocols, the type of exercise tests employed, and the methodologies used to assess mitochondrial dynamics. These findings indicate that exercise training can stimulate compensatory mechanisms via various pathways in response to the detrimental effects of imbalanced mitochondrial fusion and fission on skeletal muscle. The reaction is intended to rectify dysregulated mitochondrial dynamics and preserve muscle health. Therefore, it is imperative to restore mitochondrial dynamics to an appropriate range in atrophic skeletal muscle in order to promote muscle health and slow the progression of sarcopenia.

### 3.4. Exercise Induces Mitochondrial Redox in Sarcopenia

Muscle activity during exercise requires substantial energy from various metabolic pathways, which thus greatly relies on mitochondrial redox biology. Lactate, characterized metabolic exerkines, is a product of glycolysis and glycogenolysis. Under physical activity or exercise training, muscle continuously produces lactate and releases it into the bloodstream, and it is subsequently taken up by the liver and used in gluconeogenesis to produce glucose, which participates in the tricarboxylic acid cycle (TCA). Similarly, the catabolism of a fatty acid (beta-oxidation) during exercise is converted to fatty acetyl-CoA and then to acyl carnitine, so that it enters the mitochondria for TCA [64]. In skeletal muscle, primary aging causes defects in mitochondrial energetics and a loss of muscle mass, along with decreased physical activity and exercise [65]. Aged muscle fibers exhibit a reduced ability to utilize oxygen for the metabolism of fuels in mitochondria. Previous studies have reported diminished expressions of mitochondrial respiratory complex subunits, mitochondrial respiration, and ATP levels in skeletal muscle [66]. The accumulation of excess free radicals generated by the electron transfer chain, coupled with an impaired detoxification of ROS, contributes to cumulative damage associated with aging, ultimately compromising mitochondria through processes such as protein oxidation and mutations in mtDNA [67].

Training volume plays a crucial role in influencing changes in mitochondrial content, whereas relative exercise intensity is a key factor in determining alterations in mitochondrial respiratory function [68]. HIIT over 6 weeks improved muscle mitochondrial respiration, prompting substantial ATP regeneration by mitochondrial OXPHOS in young human skeletal muscle tissue [69]. Moreover, lifelong mixed-modality endurance exercise training may help reduce DNA methylation in the promoter regions of glycolysis, glycogen synthesis, and TCA-related genes in aged human skeletal muscle, thereby maintaining muscle mass and increasing metabolic capacity [70]. A 12-week resistance exercise training in older men (leg press and leg extension, three times/week) reversed aging-related impairments in mitochondrial ADP sensitivity, serving to further emphasize the capacity of exercise to enhance or potentially maintain mitochondrial bioenergetics in the aging process [71].

Due to the beneficial effects of mitophagy, mitochondrial biogenesis, mitochondrial dynamics, and mitochondria redox in protecting muscle, they warrant additional studies in clinical practice to identify and develop relevant therapeutic targets and strategies around exercised-induced mitochondrial homeostasis for sarcopenia.

## 4. Exerkine-Mediated Mitochondrial Homeostasis in Sarcopenia

A growing body of evidence highlights that regular exercise serves as a non-invasive therapeutic approach and a highly effective intervention for addressing metabolic syndrome linked to sarcopenia [16]. The crucial reason for this is that exercise triggers the release of bioactive factors known as exerkines, which contribute to increased muscle anabolism, bone formation, mitochondrial function, glucose utilization, and fatty acid oxidation (FAO), ultimately alleviating sarcopenia [72]. Exerkines are secreted by various tissues, including white adipose tissue (adipokines), skeletal muscle (myokines), the liver (hepatokines), and bone (osteokines).

### 4.1. The Impact of Adipokines in Sarcopenia

**Figure 3:**
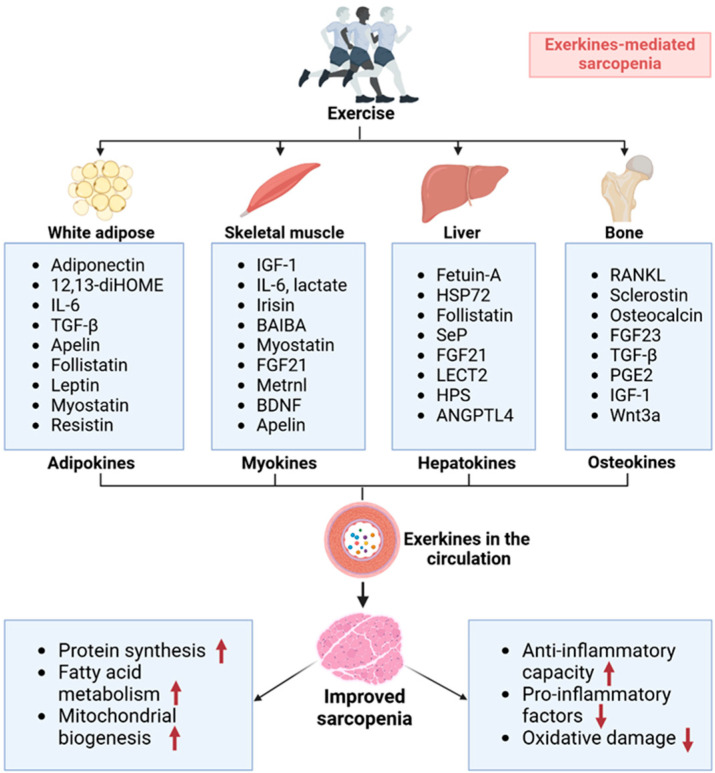
Exercise training improves sarcopenia by triggering the release of exerkines. Exerkines released after exercise into systemic circulation promote protein synthesis and mitochondrial biogenesis, increase anti-inflammatory capacity, decrease pro-inflammatory factors and oxidative damage, and enhance fatty acid metabolism to affect the skeletal muscle system and ameliorate sarcopenia, as evidenced by the adipokines (white adipose), myokines (skeletal muscle), hepatokines (liver) and osteokines (bone). 12,13-diHOME, 12,13-dihydroxy-9Z-octadecenoic acid; IGF-1, insulin-like growth factor 1; BAIBA, β-aminoisobutyric acid; FGF21, fibroblast growth factor 21; metrnl, meteorin-like factor; BDNF, brain-derived neurotrophic factor; HSP72, heat shock protein 72; SeP, selenoprotein P; LECT2, leukocyte cell-derived chemotaxin 2; HPS, hepassocin; ANGPTL4, angiopoietin-like protein 4; FGF23, fibroblast growth factor 23; PGE2, prostaglandin E2.

Presently, it is acknowledged that white adipose tissue (WAT) not only has functions in energy storage and adaptive thermogenesis but also serves as an important endocrine organ, regulating metabolism through releasing adipokines [73]. The biologically active adipokines involved in this process include adiponectin [74], Interleukin 1 (IL-1), Interleukin 6 (IL-6) [75], leptin [76], 12,13-dihydroxy-9Z-octadecenoic acid (12,13-diHOME) [77], and resistin (Figure 3) [78]. Moreover, exercise-trained WAT fosters the generation of thermogenic brown-like adipocytes with specific adipokines, including TGF-β [79], apelin, follistatin, and myostatin (Figure 3) [80,81]. Recently, great importance has been attached to the deposition of intracellular lipids, which leads to further mitochondrial dysfunction and increased ROS production in muscle, disrupts muscle protein synthesis, and impairs skeletal muscle function, causing the development of sarcopenia [82]. In light of the aforementioned characteristics, it can be considered that exercise-induced adipokines may exert a beneficial influence on sarcopenia by reducing lipid deposition.

Leptin, a key adipokine produced by adipocytes, plays a critical role in muscle metabolism modulation. It is worth noting that obesity gives rise to a state of mild inflammation, marked by the secretion of leptin, which elevates the levels of pro-inflammatory cytokines, resulting in a diminished anabolic response to IGF-1. The ensuing inflammation further contributes to insulin resistance, exacerbated by muscle catabolism, thereby leading to increased fat accumulation and muscle loss [83,84]. Studies in mice show that leptin deficiency promotes fatty acid β-oxidation, enhancing mitochondrial protection against ROS through HK-II regulation, acting on mitochondrial function and quality control mechanisms [76]. Intensive lifestyle interventions lasting 12 weeks (three times a week), including whey supplementation and resistance exercise, can significantly lower the leptin level, restore metabolic hormone levels, and ameliorate muscle mass in sarcopenic patients [85]. Transforming growth factor-beta 2 (TGF-β2), an exercise-induced adipokine, is stimulated by exercise-induced lactate, enhancing glucose uptake in skeletal muscle and boosting mitochondrial function, shedding light on adipose–muscle tissue crosstalk [79] (Table 1). Adiponectin acts through adiponectin 1 and 2 receptors (adipoR1 and adipoR2), which are highly expressed in muscle [86]. AdipoR1-deficient mice show reduced PGC-1α expression, mitochondrial biogenesis and function, and endurance training capacity [87]. A 4-month endurance training (45 min/time, 3 times/week) program in mice enhanced AdipoR1 gene expression, circulating adiponectin levels, AMPK/PGC-1α-related mitochondrial biogenesis, and Akt/mTOR-mediated protein synthesis and proliferation, showcasing positive effects on sarcopenia development and muscle health [74] (Table 1). Exercise-induced adiponectin also reduces white fat synthesis to mitigate muscle atrophy, with the primary mechanisms underlying this effect including enhanced insulin sensitivity, improved glucose uptake, promotion of myogenesis, inhibition of muscle protein degradation, and increased muscle hypertrophy [87,88]. Additionally, adiponectin contributes to the activation of brown adipose tissue (BAT) and promotes thermogenesis, further supporting the preservation of muscle mass and overall metabolic health [89].

Therefore, according to the above studies, exercise-induced adipokines are vital in reducing inflammation, promoting mitochondrial biogenesis, and decreasing white fat synthesis to attenuate sarcopenia. Understanding the specific adipokines activated by exercise can guide the development of more effective exercise interventions and therapeutic strategies for improving skeletal muscle health.

### 4.2. The Impact of Myokines in Sarcopenia

Consistent research findings highlight the significant health benefits of regular exercise, contributing to reduced age-related oxidative damage, enhanced autophagy, and improved mitochondrial function, myokine profiles, IGF-1 signaling pathways, and insulin sensitivity [96]. The intricate mechanisms underlying these health advantages are multifaceted, partly attributable to the release of bioactive substances in skeletal muscle during exercise. Skeletal muscle operates as an endocrine organ, producing and releasing myokines that exert autocrine, paracrine, and hormonal influences on various tissues [97], including IGF-1 [90], IL-6 [91], irisin [98], β-aminoisobutyric acid (BAIBA) [99], myostatin [100], fibroblast growth factor 21 (FGF21) [101], meteorin-like factor (metrnl) [102], brain-derived neurotrophic factor (BDNF) [103], and apelin [13] (Figure 3). Dysregulation in myokine secretion can arise in the pathogenesis of age-related conditions, such as sarcopenia [104,105].

#### 4.2.1. IGF-1

IGF-1, a key factor controlling skeletal muscle anabolism and catabolism, exerts its anabolic effects on skeletal muscle by regulating mitochondrial function, ROS detoxification, and the basal inflammatory state in aging individuals [106]. However, IGF-1 levels are inhibited in numerous chronic diseases, including sarcopenia, potentially leading to muscle atrophy resulting from the combined impacts of altered protein synthesis, ubiquitin-proteasome system (UPS) activity, autophagy, and impaired muscle regeneration [107]. One study result demonstrated that aerobic exercise (12 m/min for 60 min daily), resistance exercise (60 min daily), whole-body vibration (15 min daily), and electrical stimulation (15 min daily) over 4 weeks inhibited oxidative stress and apoptosis and enhance skeletal muscle mass and function in mice undergoing early aging by activating the IGF-1/IGF-1R–PI3K/Akt signaling pathway [90] (Table 1).

#### 4.2.2. BDNF

Muscle-derived BDNF serves as a new regulator of mitochondrial remodeling in skeletal muscle in response to lipid overload [108]. BDNF promotes the mitochondrial transport of fatty acids and the expression of genes related to FAO by activating the AMPK, PPARα, Nur77, and PGC-1α pathways [109]. Since these signaling pathways are also critical for mitochondrial biogenesis and autophagy during fasting, their disruption in muscle with muscle-specific BDNF knockout leads to a reduction in ATP production through FAO, as well as diminished amino acid release from fasting-induced autophagy, resulting in myofiber necrosis [109]. Impaired muscle fibers are thus associated with weakened muscle strength, reduced physical activity, lower metabolic rate, and increased adiposity.

#### 4.2.3. IL-6

IL-6 also acts as a major myokine with anti-inflammatory and anabolic effects. Circulating IL-6 concentrations increase dramatically in response to exercise and are intricately tied to exercise intensity, meaning that IL-6 is released from the skeletal muscle to restore muscle function following exercise [110]. An acute exhaustive treadmill exercise (20 m/min for 90 min) triggered increased mitochondrial respiration and mitochondrial biogenesis in mice, suggesting that the beneficial adaptations of physical exercise, such as enhanced mitochondrial content and improved autophagy, may be driven by the interaction between IL-6 and REVERBα [91] (Table 1).

#### 4.2.4. Irisin

Irisin is an exercise-induced PGC-1α-dependent myokine, with significant links to various aging-related diseases, including sarcopenia. The stimulation of exercise and cold exposure enhance the generation and secretion of irisin from skeletal muscle, thereby inducing myogenesis, preventing muscle atrophy, improving energy metabolism, enhancing cellular homeostasis, and promoting mitochondrial quality control [98]. Serum levels of irisin could be used as a biomarker of muscle dysfunction. Resistance exercise for 12 weeks (ladder climbing exercise three times a week in mice; elastic band exercise program consisting of 1 h sessions twice a week for older adults) elevated irisin levels and improved muscle strength, which might present an effective strategy to counteract age-related decline in muscle function [92] (Table 1).

#### 4.2.5. Myostatin

Myostatin is a powerful inhibitor of muscle mass growth and development that acts via Akt/TORC1/p70S6K signaling, inhibiting myoblast differentiation and reducing myotube size [111]. It has been found that the expression of myostatin can be influenced by factors such as exercise, NF-κB, aging, and TNF-α. Myostatin is increased in patients with muscle loss, and its deficiency increases muscle cell protein synthesis, reduces degradation, promotes mitochondrial biogenesis, and preserves muscle function [100]. An eight-week concurrent training (resistance and endurance training, three training sessions per week) reduced myostatin and improved body composition, muscle mass, and function in sarcopenic elderly men [112].

#### 4.2.6. FGF21

FGF21 is acknowledged as a marker of mitochondrial dysfunction and aging, playing a crucial role in regulating metabolic activity [113]. It has been reported that FGF21 may exert its effects on muscle mass via regulation of the mitophagy protein Bnip3 [101]. The expression level of FGF21 is very low in normal healthy muscle. However, muscle FGF21 is released under conditions including fasting, exercise, mitochondrial dysfunction, mitochondrial myopathy, and metabolic disorders [114]. During exercise (e.g., resistance training), FGF21 is released from muscles into the bloodstream to optimize mitochondria that act as an energy generator, thus preventing certain diseases, especially sarcopenia [115].

#### 4.2.7. Lactate

Lactate, described as a myokine, exists in millimolar concentrations in muscle and other tissues. In response to physical activity or exercise training, muscle constantly produces lactate and releases into the bloodstream. Lactate crosses membranes through several monocarboxylate transporters (MCTs) in skeletal muscle. In particular, MCT1 is associated with lactate intake from the circulation and muscle oxidation capacity. Exercise training increases MCT1 levels and enhances lactate transport to muscle, whereas MCTs are reduced in aging muscle, thus reducing lactate levels and increasing skeletal muscle fatigue [116]. In mice, elevated lactate decreased the number of apoptotic nuclei in aged muscle and restored mitochondrial function through the activation of the CREB-PGC-1α pathway, thereby helping to mitigate sarcopenia [117].

Overall, exercise-induced myokines from the activation of signaling pathways reduce muscle protein degradation, promote protein synthesis and hypertrophy, and maintain mitochondrial homeostasis to prevent sarcopenia. Nonetheless, the periodicity and dynamic changes in myokines’ metabolic adaptations upon different types of exercise training and their contribution in clinical application require further investigation.

### 4.3. The Impact of Hepatokines in Sarcopenia

Hepatokines represent a novel class of exercise-inducible soluble factors released by the liver during and after physical activity, including selenoprotein P (SeP) [93], FGF21 [118], heat shock protein 72 (HSP72) [119], and hepassocin (HPS) (Figure 3) [120]. These hepatokines are believed to play a pivotal role in fostering interactions among various organs, such as skeletal muscle, adipose tissue, and the central nervous system (CNS), exerting tissue-protective effects that counteract oxidative stress, inflammation, mitochondrial dysfunction, and apoptosis [121]. For example, hepatokines such as FGF21, angiopoietin-like protein 4 (ANGPTL4), and follistatin, released during and after exercise, may influence skeletal muscle metabolism to favor redistribution [120].

Fetuin-A, a systemic calcification inhibitor, induces a pro-inflammatory milieu by polarizing M1 macrophages in WAT, generating ROS and reducing insulin sensitivity, which combined with other pro-inflammatory cytokines causes the development of sarcopenia [122,123,124,125]. Of new note, SeP is identified as a hepatokine upregulated in aging, and it causes signaling resistance via reductive stress, which is involved in physical inactivity-mediated muscle atrophy [126]. Following exercise training on a treadmill (30 min per day for 4 weeks), SeP-deficient mice exhibited an increased aerobic exercise capacity accompanied by increased mitochondrial biogenesis and function in skeletal muscle, an effect associated with increased ROS, AMPK activation, and increased PGC-1α activity [93] (Table 1). Follistatin, a versatile regulatory protein primarily secreted by the liver, serves as a potent inhibitor of myostatin-mediated sarcopenia. Transgenic mice overexpressing follistatin that followed a voluntary wheel running (speed >0.2 km/day) program for 5 months showed a notable increase in muscle mass and contractility [94] (Table 1).

In summary, these studies highlight the potential of hepatokines and their associated signaling pathways as promising therapeutic targets for managing sarcopenia. Their role in regulating mitochondrial homeostasis underscores their importance in developing new treatment strategies for this condition.

### 4.4. The Impact of Osteokines in Sarcopenia

Osteokines, novel exercise-inducible soluble factors secreted by osteocytes, osteo blasts, and osteoclasts, play an important role in the body’s response to exercise, mainly including RANKL [127], sclerostin [128], osteocalcin [129], fibroblast growth factor 23 (FGF23) [95], Wnt3a [130], TGF-β [131], IGF-1 [132], and prostaglandin E2 (PGE2) [133] (Figure 3). Osteokines are essential for regulating the expression of PGC-1α, improving insulin sensitivity, and promoting mitochondria biogenesis in skeletal muscle [134]. The muscle–bone crosstalk may represent a key point in the development of osteosarcopenia (a musculoskeletal disorder) therapies [135].

The principal products secreted from bone that influence the formation, maintenance and regeneration of muscle are the osteocalcin, a marker protein of osteoblasts, and FGF23, a phosphatonin derived from osteocytes, as well as the inhibitors of the osteogenic wingless (Wnt) signaling pathway, sclerostin [136]. Osteocalcin, mainly synthesized and released by osteoblasts, is involved in increasing muscular mitochondria, inhibiting myoblast proliferation and bone formation, and regulating energy homeostasis, known to increase during 8-week treadmill running (65–75% VO_2_max per session, four sessions per week) in obese young males [137]. In older mice, osteocalcin signaling in the myotubes promotes protein synthesis without affecting protein breakdown via activation of the mTOR pathway, uncovering that osteocalcin is both necessary and sufficient to attenuate age-related muscle loss [129]. RANKL and its associate RANK act in an upstream signaling pathway of nuclear factor-κB (NF-κB). Research has shown that RANKL inhibition improves muscle strength and insulin sensitivity in osteoporotic mice and humans through the RANK/RANKL/OPG signaling pathways, potentially representing a new therapeutic avenue for sarcopenia [127]. FGF23, a unique hormone-like osteokine secreted by bone cells, has been shown to influence muscle performance. A single bout of acute exercise (60 min), exhaustive exercise, and chronic prolonged exercise (60 min per day for one week) in mice stimulated serum FGF23 levels to promote muscle performance by regulating ROS production and modulating mitochondrial function in skeletal muscle [95] (Table 1).

After understanding the cross-talk between bone and muscle, further research should focus on identifying specific targets involved in exercise-induced osteokine protection against sarcopenia, which might pave the way for novel treatments of sarcopenia and other muscle disorders.

## 5. Conclusions and Future Prospects

Exercise can induce significant adaptations in skeletal muscle, including increased muscle mass, enhanced muscle regeneration and hypertrophy, reduced ROS, elevated the release of hormones, and improved mitochondrial function. Very importantly, an increasing number of exerkines released during exercise present the potential to induce mitophagy, mitochondrial biogenesis, dynamics, autophagy, and redox to enhance mitochondrial homeostasis, thereby maintaining the skeletal muscle metabolic balance and mitochondrial health. Understanding these mechanisms could be pivotal in identifying exerkines as key protective sensors of exercise to guide more effective and safer exercise regimens for sarcopenia patients and to predict their prognosis. However, severe sarcopenia patients are characterized by diminished exercise tolerance and may not be able to withstand extended exercise training periods. Besides exercise therapy, it is therefore imperative to investigate novel therapeutic strategies for the management of sarcopenia in the future. The complex interactions between exerkines and other tissues (e.g., adipose tissue, liver, bone, etc.) and the skeletal muscle by the regulation of mitochondrial homeostasis warrants further investigation. The therapeutic effects based on exerkine-regulated molecules, especially mitochondrial homeostasis, need to be evaluated in animal models and further translated into human treatments.

## Figures and Tables

**Table 1:** The effects of exerkines in sarcopenia.

Exerkines	Main Tissue of Origin	Main Mechanism	Main Biological Action	Exercise Type	Exercise Protocol	Organism or Model	Refs
TGF-β2	AT	Lactate–TGF-β2	Regulates glucose and fatty acid metabolism; promotes mitochondrial function	Voluntary wheel running (mice); cycling (humans)	11 or 28 d (mice); 12 weeks, 5 d/week, 60–80 min/d (humans); 2 weeks, 6 sessions, 4–6 bouts for 30 s/session, separated by 4 min (humans)	Mice and humans	[79]
Adiponectin	AT	Adiponectin/AdipoR1-AMPK-PGC-1α	Restores muscle stem cell mobilization, alters muscle metabolic, and stimulates mitochondrial biogenesis	Treadmill running	4 months, 18 m/min for 35 min, 3 times/week	Mice	[74]
IGF-1	SkM	IGF-1/IGF-1R-PI3K/Akt	Mediates autophagy and protein synthesis	Treadmill training, ladder-climbing training, vibration exercise training, electrical stimulation	4 weeks, 60 min/d; 4 weeks, 60 min/d; 4 weeks, 15 min/d; 4 weeks, 15 min/d	Mice	[90]
IL-6	SkM	REVERBα/IL-6-STAT3	Increases mitochondrial respiration and mitochondrial biogenesis	Treadmill running	90 min, 20 m/min	Mice	[91]
Irisin	SkM	FGF21-PGC-1α -Irisin	Improves muscle strength and function	Ladder climbing exercise with tail weight (aged mice); elastic band exercise(humans)	12 weeks, 3 d/week (aged mice); 12 weeks, 1 h session, 2 d/week (humans)	Aged mice, and humans aged over 65 years	[92]
SeP	Liver	SeP/AMPK/PGC-1α	Enhances mitochondrial biogenesis and function	Treadmill training	4 weeks, 5 d/week, 30 min/d	Mice	[93]
Follistatin	Liver	Follistatin/Myostatin	Regulates metabolic	Voluntary wheel running	5 months, speed >0.2 km/d	Mice	[94]
FGF23	Bone	FGF23-Klotho	Promotes muscle performance and enhances mitochondrial function	Motor treadmill exercise	5 m/min for 10 min, +5 m/min → 20 m/min, acute (60 min), exhaustive, and moderately chronic (one week, 60 min/d) exercise	Mice	[95]

TGF-β2, transforming growth factor-beta 2; d, day(s); HR, heart rate; adipoR1, adiponectin receptor1; AT, adipose tissue; SkM, skeletal muscle; AMPK, AMP-activated protein kinase; PI3K; phosphatidylinositol 3-kinase; Akt, protein kinase B; PGC-1α, peroxisome proliferator-activated receptor-γ coactivator-1α; IL-6, Interleukin 6; STAT3, signal transducer and activator of transcription 3; FGF21, fibroblast growth factor 21; IGF-1, insulin-like growth factor 1; SIT, sprint interval training; SeP, selenoprotein P; FGF23, fibroblast growth factor 23; +5m/min → 20m/min, increased by 5 m/min until 20 m/min.

## Data Availability

No new data were created or analyzed in this study.
